# Matrix stiffness regulates myocardial differentiation of human umbilical cord mesenchymal stem cells

**DOI:** 10.18632/aging.202244

**Published:** 2020-12-09

**Authors:** Yingying Sun, Jingwei Liu, Ziran Xu, Xiaoxuan Lin, Xiaoling Zhang, Lisha Li, Yulin Li

**Affiliations:** 1 Department of Stomatology, The First Hospital of Jilin University, Jilin University, Changchun, China; 2 The Key Laboratory of Pathobiology, Ministry of Education, College of Basic Medical Sciences, Jilin University, Changchun, China; 3 College of Clinical Medicine, Jilin University, Changchun, China; 4 Key Laboratory of Organ Regeneration and Transplantation of Ministry of Education, The First Hospital, Jilin University, Changchun, China; 5 National-Local Joint Engineering Laboratory of Animal Models for Human Diseases, Changchun, China

**Keywords:** human umbilical cord mesenchymal stem cells, matrix stiffness, myocardial differentiation, Piezo1, integrin β1

## Abstract

Myocardial infarction is a cardiovascular disease with high mortality. Human umbilical cord mesenchymal stem cells (hUC-MSCs) with strong self-renewal capacity and multipotency, provide the possibility of replacing injured cardiomyocytes. hUC-MSCs were cultured on polyacrylamide hydrogels with stiffnesses corresponding to Young's modulus of 13-16kPa and 62-68kPa which mimic the stiffnesses of healthy heart tissue and fibrotic myocardium. The expression of early myocardial markers Nkx2.5, GATA4, Mesp1 and the mature myocardial markers cTnT, cTnI, α-actin were detected by RT-PCR and Western Blot, which showed that soft matrix (13-16 kPa) tended to induce the differentiation of hUC-MSCs into myocardium, compared with stiff matrix (62-68 kPa). Piezos are mechanically sensitive non-selective cation channels. The expression of Piezo1 increased with the stiffness gradient of 1-10kPa, 13-16kPa, 35-38kPa and 62-68kPa on the 1^st^ day, but Piezo2 expression was irregular. The expression of integrin β1 and calcium ions were also higher on stiff substrate than on soft substrate. hUC-MSCs tend to differentiate into myocardium on the matrix stiffness of 13-16 kPa. The relationship among matrix stiffness, Piezo1 and myocardial differentiation needs further validation.

## INTRODUCTION

Myocardial infarction has been the leading cause of sudden cardiac death worldwide. The incidence rate increases by estimated 41% for ST segment elevation myocardial infarction [1] and 61% for non-ST-elevation myocardial infarction [2] per 5-year increase in age. Myocardial fibrosis is the common pathological feature of myocardial infarction at the final stage with a collagen-based scar resulting from the excessive accumulation of extracellular matrix (ECM). The matrix stiffness changes during the repair process of myocardium [3].

At present, the main treatment of cardiovascular disease is drug treatment and surgical intervention, but the symptoms can only be alleviated partially. Once cardiomyocytes are damaged, irreversible loss will occur due to the low regenerative capacity of human adult cardiomyocytes. A significant clinical need of cardiomyocytes exists for new drug development *in vitro* or cell-based therapies for heart repair *in* *vivo*.

Mesenchymal stem cells (MSCs) have the capacity to self-renew and to develop into multiple specialized cell types present in a specific tissue or organ. They can home to the injured tissue actively and participate in the repair and immunoregulation [4]. MSCs have the potential to differentiate into three germ layers: they can be induced into cells from mesoderm, such as cardiomyocytes [5], osteoblasts, adipocytes [6], vascular endothelial cells [7]; from endoderm, such as epithelial cells and lung cells [8]; and from ectoderm, such as neurocytes [9].

Human umbilical cord mesenchymal stem cells (hUC-MSCs) can be isolated from the Wharton's Jelly tissue of the umbilical cord [10, 11], with the advantages of turning waste into treasure, non-invasive extraction and no ethical controversy. In the treatment of myocardial infarction by transplantation of stem cells, both umbilical cord mesenchymal stem cells (hUC-MSCs) and bone marrow mesenchymal stem cells (BM-MSCs) can improve cardiac function and inhibit remodeling of myocardium [12]. hUC-MSCs are expected to replace BM-MSCs as a better research object in cell therapy [13].

At present, common methods of differentiation towards cardiomyocytes include chemical reagents and biological factors, which are conducted on rigid plastic petri dishes. But chemical reagents may have carcinogenic risks, and biological factors are relatively expensive. Furthermore, the cardiomyocytes obtained by these methods differ from adult mature cardiomyocytes in structure, proliferation, metabolism and electrophysiology, better approximating fetal cardiomyocytes [14]. Therefore, it is necessary to find new ways to solve this problem.

ECM is not only an important part of the body, but also one of the main factors that make up the cellular micro-environment. It guides the behavior of cells through the surface roughness and stiffness of the matrix, so as to regulate the biological behavior such as cell growth, differentiation, migration and proliferation [15–19]. Different matrix stiffness has different effects on cell fate. Matrix stiffness can determine the self-renewal and multipotency of MSCs [20–26].

To imitate the growth environment of cells under physiological conditions *in vitro*, polyacrylamide hydrogels were used in 2006 by Engler et al. It confirmed ECM stiffness can guide the differentiation of MSCs for the first time. Since then many synthetical alternatives for ECM have emerged, such as polymer material polystyrene, collagen hydrogels, collagen-hyaluronic acid composite and polydimethylsiloxane [26]. Many experiments have proved that matrix stiffness as an independent factor instructed the maturation of the already differentiated cardiomyocytes and the induction and proliferation of cardiomyocytes from undifferentiated progenitors [27–30].

It is necessary to find the appropriate stiffness for hUC-MSCs differentiation into myocardium. During growth of the heart, the stiffness of its surrounding ECM is dynamic. The matrix stiffness of the epicardium increased three times during development, while the matrix stiffness of the fibrosis scar formed after myocardial infarction was 3-4 times as the surrounding normal myocardial tissue [26, 31]. The change of elastic modulus of myocardial tissue can regulate its function. Matrix stiffness not only affects the active contractility produced in myocardium, but also influences the contractile strain, and has a significant effect on the beating frequency of cardiomyocytes. Many experiments have proved that the appropriate matrix stiffness is the key to induce stem cells differentiating into cardiomyocytes [32–34].

How does matrix stiffness direct the growth and differentiation of MSCs? Piezos are mechanically sensitive non-selective cation channels in mammalian cells [35], which senses changes in the mechanical force of the cell membrane and reacts rapidly. The ion channel can transform the mechanical signal into an electrical or chemical signal, which can be activated by pressure. The evolutionarily conserved Piezo family of proteins include Piezo1 and Piezo2, containing no sequence homology with any known class of ion channels [36].

Integrins are a class of noncovalent related heterodimeric transmembrane receptors, consisting of two subunits α and β. As a mechanical signal receptor on the cell membrane, integrin extracellular region binds to the ligand in the ECM to control adhesion attraction, and its intracellular region binds to the cytoskeleton rapidly, which leads to the formation of focal adhesions. The changes in the extracellular environment are sensed dynamically through the force created by adhesion attraction. The changes in integrin conformation can adjust the cytoskeleton and then influence cell morphology and mechanical properties [37]. Integrins are sensitive to matrix stiffness [38].

Calcium ion (Ca^2+^) as a second messenger can regulate the transcription of numerous phenotypic genes and transcription factors and control cellular biological functions, especially electrophysiological functions. Ca^2+^ plays its important role through the changes of cytoplasmic Ca^2+^ concentration [39].

When exploring the relationship among Piezo1, integrins and Ca^2+^, it was found that the Fam38A (Piezo1) siRNA knockdown in epithelial cells inactivates endogenous integrin β1, reducing cell adhesion [40]. Piezo1 localizes at focal adhesions to activate integrin-FAK signaling, regulate extracellular matrix, and reinforce tissue stiffening. In turn, a stiffer mechanical microenvironment elevates Piezo1 expression [41]. The cytoskeleton binding to Integrin makes it more difficult to open the channel, which protects the Piezo1 mechanically. And knocking down the filamin protein, the scaffold protein between actin and membrane protein, can activate Piezo1 more easily detected by patch clamp [42]. The mechanical force that activates Piezo1 arises from Myosin II phosphorylation by Myosin Light Chain Kinase [43]. Activation of Piezo1 increased Ca^2+^ influx and inhibited Notch signaling pathways. It indicates that calcium ions and Piezo1 are closely linked during differentiation and development of cells [44]. In conclusion, Piezo1 is activated by the traction produced by myosin along the focal adhesion with rich Integrins of the actin cytoskeleton.

The results of this project will greatly contribute to better understanding of the role ECM plays in the growth and differentiation of MSCs, and accelerate the use of hUC-MSCs in clinical treatments of myocardial infarction.

## RESULTS

### Culture and characterization of hUC-MSCs

**Figure 1:**
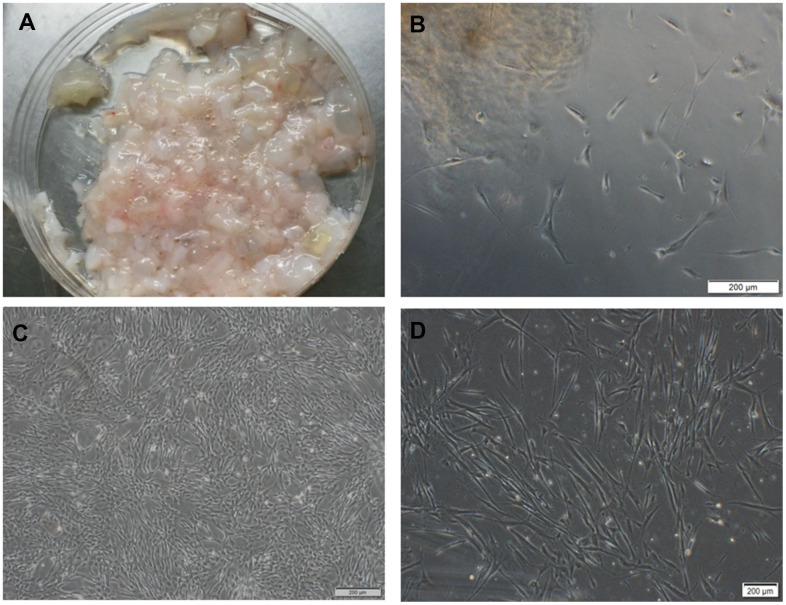
**hUC-MSCs isolated from Wharton's Jelly tissue.** (**A**) Wharton's Jelly tissue. (**B**) A small number of cells crawled out of the tissue block on the 5^th^ day, varying in morphology. Scale bar=200 μm. (**C**) P5 cells in the shape of vortex or shoal. Scale bar=200 μm. (**D**) P6 cells after cryopreservation and resuscitation. Scale bar=200 μm.

With the informed consent of the pregnant and approval by the ethics committee, we got the umbilical cord of a full-term fetus from the First Affiliated Hospital of Jilin University. The primary cells were isolated from Wharton's Jelly tissue by tissue culture method (Figure 1A). A small number of cells varying in morphology crawled out of the tissue block from the 5^th^ day (Figure 1B) to the 10^th^ day. As time went by, the cells principally formed bipolar spindle-like cells after they grew to passage 3. When confluence reached 90%, the cells exhibited a spiral shape (Figure 1C, 1D).

**Figure 2:**
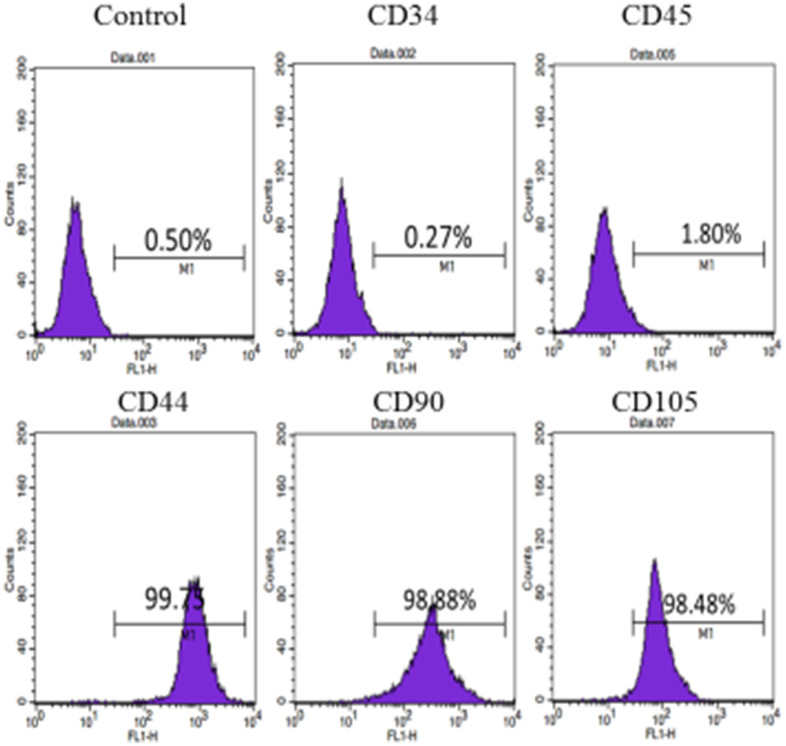
**Cell surface marker identification of hUC-MSCs: Cellular immunophenotype detected by flow cytometry.** The results show that the cells express CD44, CD90 and CD105 positively, and CD34 and CD45 negatively. n=3.

Flow cytometry were performed on the obtained cells. The cells expressed CD44, CD90 and CD105 positively, and expressed CD34 and CD45 negatively, indicating that the cells had the characteristics of mesenchymal stem cells (Figure 2).

**Figure 3:**
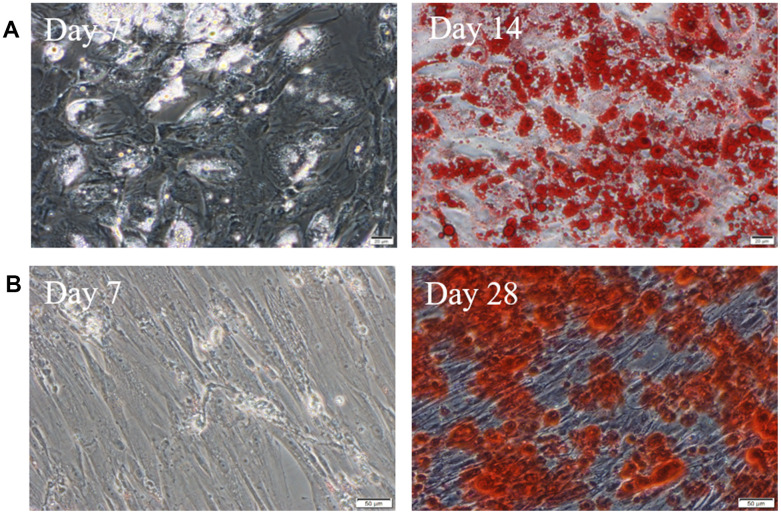
**hUC-MSCs induced into adipocytes and osteoblasts.** (**A**) Induced into adipocytes for 7 days and 14 days. The lipid droplets in cells were stained by Oil Red O. Scale bar=50μm. (**B**) Induced into osteoblasts for 7 days and 28 days. There are bright red calcium nodules stained by alizarin red in cytoplasm. Scale bar=50μm. n=3.

After 1 week of adipogenic induction, the shape of cells changed from spindle into column, and a few lipid droplets stained by Oil Red O emerged. After 2 weeks of adipogenic induction, the cells gradually became oval, and the cytoplasm was filled with vacuoles of oil droplets stained in red (Figure 3A). During osteogenesis induction, the cells gradually changed from spindle to polygon. After 4 weeks, there are bright red calcium nodules stained by alizarin red (Figure 3B).

The hUC-MSCs isolated from different human subjects (n=4) showed no significant differences in morphology, surface marker, and multipotency.

### Matrix stiffness regulates cellular morphology

**Figure 4:**
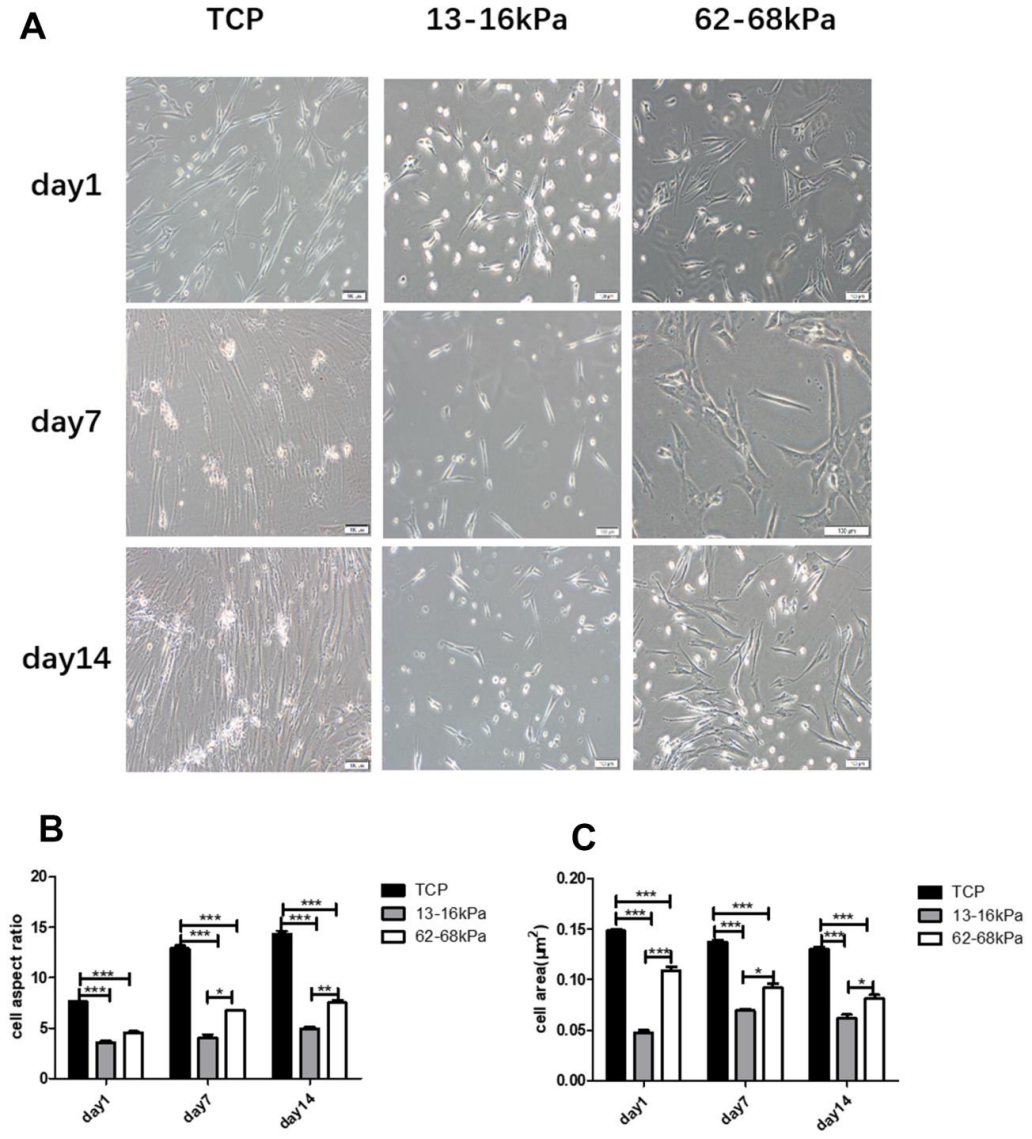
**Matrix stiffness regulates the cellular morphology.** (**A**) hUC-MSCs were cultured on matrix gels with different stiffness of 13-16kPa and 62-68 kPa. Compared with the control group, more cells on 13-16kPa were shaped like round dots on the 1^st^ day, and like short columnar cells on the 7^th^ day. On the 14^th^ day, the morphology of cells was like cardiac muscle cells. Cells on 62-68 kPa were in the shape of polygon on the 1^st^ day, and were in the shape of long spindle on the 7^th^ day and the 14^th^ day. Scale bar = 100μm. (**B**) Aspect ratio of each group. (**C**) Area of each group. Both aspect ratio and area of cells on matrix of 13-16kPa were the lowest, and the control were the largest within them. *P<0.05, **P<0.01, ***P<0.001. n=3.

According to previous studies and experiments, we prepared PAAm hydrogels with different stiffness of 13-16 kPa and 62-68 kPa to imitate the stiffness of myocardium and fibrotic scar. hUC-MSCs were cultured on the different matrix gels for 1 day, 7 days and 14 days, whose morphology has changed significantly. Compared with the control group, more cells on 13-16kPa were shaped like round dots on the 1^st^ day, and like short columnar cells on the 7^th^ day. On the 14^th^ day, the morphology of cells was like myocardial cells. Cells on 62-68 kPa were in the shape of polygon on the 1^st^ day, and were in the shape of long spindle on the 7^th^ day and the 14^th^ day (Figure 4A). Then we measured the aspect ratio and area of the cells, which of cells on matrix of 13-16kPa were the lowest, and which of cells on matrix of 62-68kPa were the second lowest. The aspect ratio and area of control group cells were the largest (Figure 4B, 4C).

### Expression of myocardial markers on mRNA and protein level

**Figure 5:**
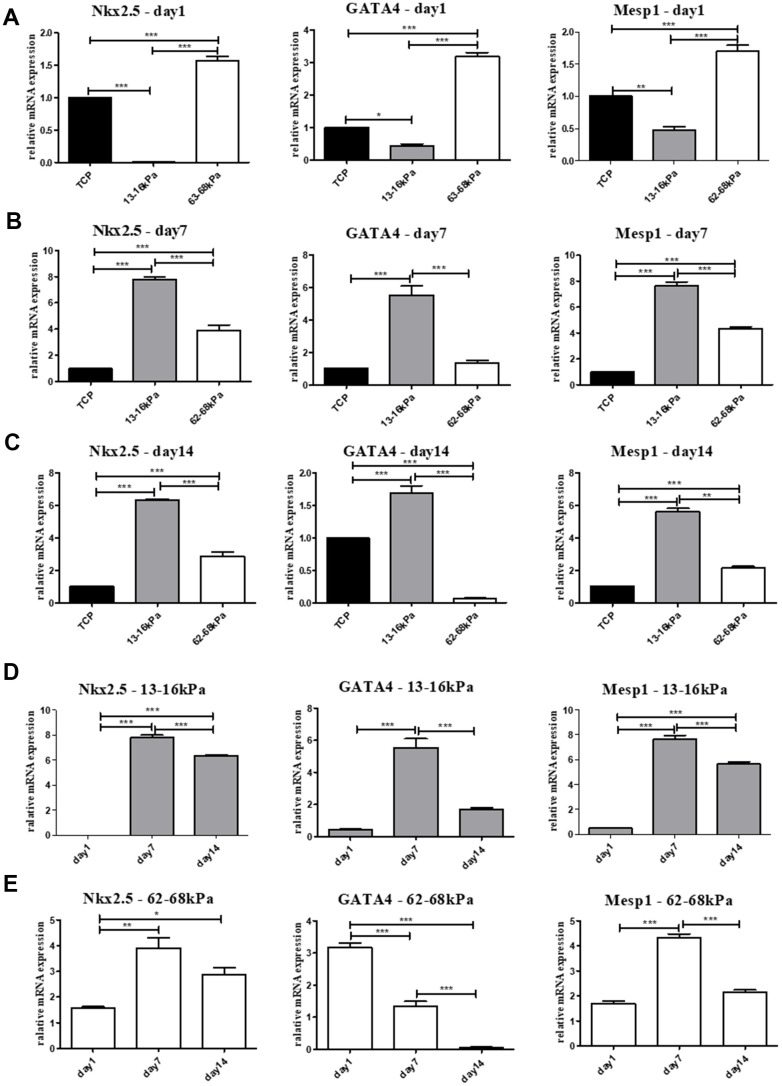
**Expression of early myocardial markers on mRNA level.** (**A**–**C**) The relative mRNA expression of early myocardial markers Nkx2.5, GATA4 and Mesp1 on different matrix of 13-16 kPa, 62-68 kPa and control group at the same time point, the 1^st^ day, the 7^th^ day and the 14^th^ day. On the 7^th^ day and the 14^th^ day, the expression on the 13-16 kPa matrix was highest among the three groups. (**D**, **E**) The expression tendency of the three early myocardial markers on the same stiffness changed with time. The highest expression of Nkx2 and Mesp1 on 62-68 kPa and 13-16 kPa emerged on the 7^th^ day, while the expression of GATA4 decreased gradually over time. *P<0.05, **P<0.01, ***P<0.001. n=3.

The mRNA expressions of early myocardial markers Nkx2.5, GATA4, Mesp1 and mature myocardial markers cTnT, cTnI, α-actin were detected by qRT-PCR. On the 1^st^ day, the expression of early myocardial markers on 13-16 kPa were lower than 62-68 kPa, may because cells were more sensitive to the stiff matrix in a short time (Figure 5A). On the 7^th^ day and the 14^th^ day, cells grew stably on the matrix, and the expression of early myocardial markers on the 13-16 kPa matrix was highest among the three groups (Figure 5B, 5C). The expression of the three early myocardial markers on the same stiffness at different time points showed that the expression of Nkx2.5, GATA4 and Mesp1 on 13-16 kPa were the highest on the 7^th^ day, followed by 14^th^ day (Figure 5D). The condition of Nkx2 and Mesp1 on 62-68 kPa were like that on 13-16 kPa, whose highest expression emerged on the 7^th^ day, while the expression of GATA4 decreased gradually over time (Figure 5E).

**Figure 6:**
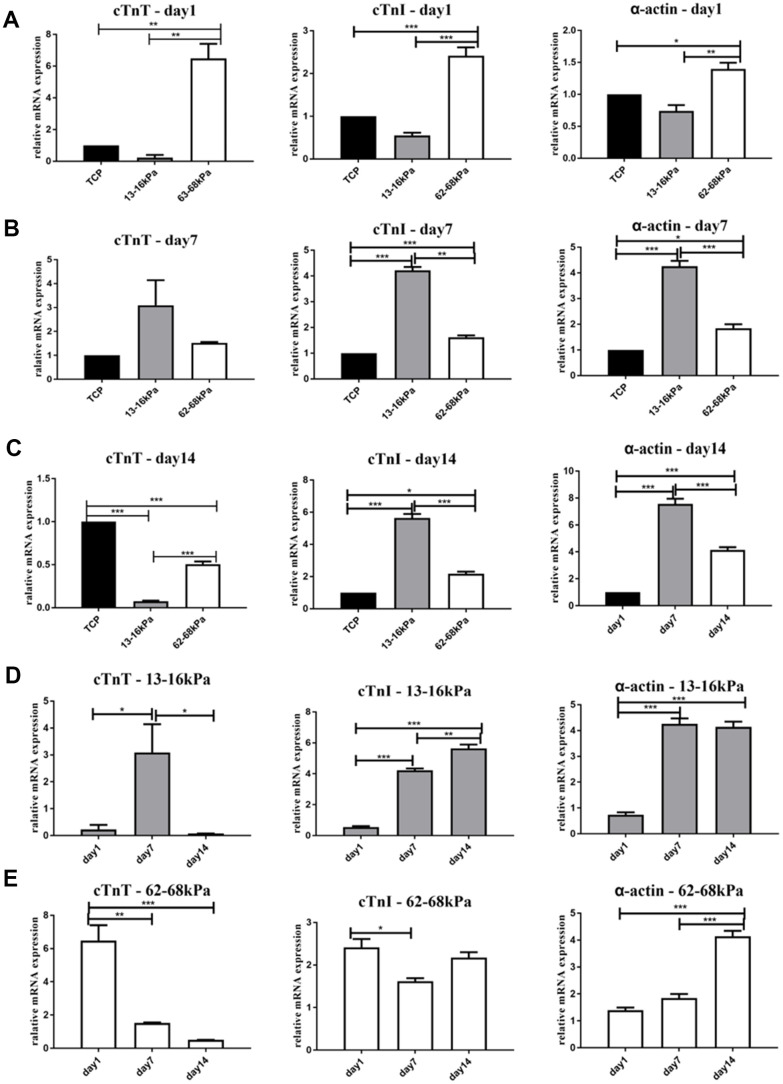
**Expression of mature myocardial markers on mRNA level.** (**A**–**C**) The relative mRNA expression of mature myocardial markers cTnT, cTnI and α-actin on different matrix of 13-16 kPa, 62-68 kPa and control group at the same time point, the 1^st^ day, the 7^th^ day, and the 14^th^ day. On the 7^th^ day and the 14^th^ day, the expression of cTnI and α-actin on 13-16 kPa matrix were the highest. The cTnT on 13-16 kPa was expressed the most on the 7^th^ day, but its expression decreased on the 14^th^ day. (**D**, **E**) The expression of the three mature myocardial markers on the same stiffness at different time points. The expression of cTnI and α-actin increased over time on 13-16 kPa matrix, and the expression of cTnT rose to the highest on the 7^th^ day but decreased on the 14^th^ day. On the 62-68 kPa matrix, the expression of α-actin increased over time, while the expression of cTnT and cTnI in the matrix stiffness groups were lower than that of the control group. *P<0.05, **P<0.01, ***P<0.001. n=3.

On the 1^st^ day, the expression of mature myocardial markers on 13-16 kPa were lower than 62-68 kPa (Figure 6A). On the 7^th^ day, the expression of three mature myocardial markers on 13-16 kPa matrix were the highest (Figure 6B). On the 14^th^ day, the expression of cTnI and α-actin on 13-16 kPa were still the highest, while the expression of cTnT decreased in the soft matrix group (Figure 6C). The expression of these mature myocardial markers on the same stiffness at different time points showed that cTnI and α-actin increased over time on 13-16 kPa matrix, and the expression of cTnT rose to the highest on the 7^th^ day but decreased from the 14^th^ day (Figure 6D). On the 62-68 kPa matrix, the expression of α-actin increased over time, while the expression of cTnT and cTnI on the matrix stiffness groups were lower than that of the control group (Figure 6E).

**Figure 7:**
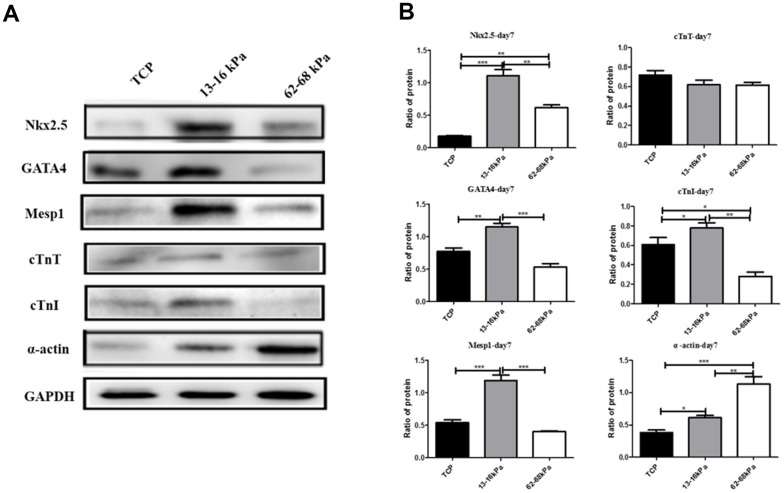
**Expression of myocardial markers on protein level.** (**A**) The photograph of Western Blot detected the expression of Nkx2.5, GATA4, Mesp1, cTnT, cTnI and α-actin on protein level. (**B**) Graph of the Western Blot. *P<0.05, **P<0.01, ***P<0.001. Nkx2.5, GATA4, Mesp1, and cTnI were all highly expressed on 13-16 kPa matrix, compared with 62-68kpa and control group. n=3.

Western Blot was used to detect the protein levels of myocardial markers Nkx2.5, GATA4, Mesp1, cTnT, cTnI and alpha-actin. The total proteins of hUC-MSCs cultured on matrix with different stiffness were collected on the 7^th^ day. Compared with the control group, Nkx2.5, GATA4, Mesp1, cTnT, cTnI and alpha-actin were all highly expressed on 13-16 kpa matrix. Compared with 62-68kpa, Nkx2.5, GATA4, Mesp1 and cTnI were all highly expressed on 13-16kpa matrix, which was consistent with qPCR results, further proving that hUC-MSCs tended to differentiate myocardium on 13-16kpa matrix (Figure 7A, 7B).

### Matrix stiffness influences Piezo1 expression

**Figure 8:**
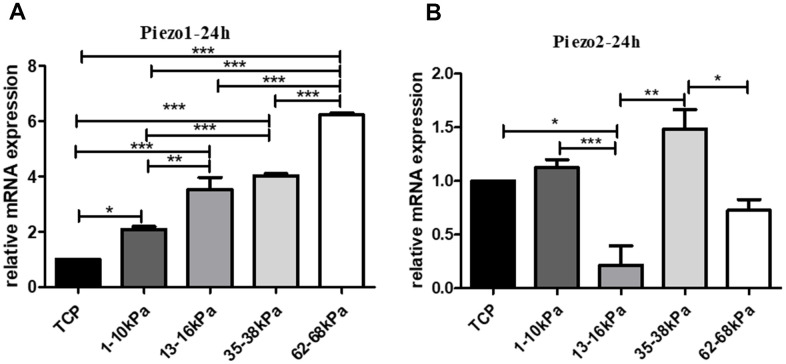
**Expression tendency of Piezo on matrix with different stiffness.** (**A**) The expression of Piezo1 on different matrix with stiffness in grade. (**B**) The expression of Piezo2 in the same condition. QPCR results showed that Piezo1 increased in stiffness-dependent mode, and Piezo2 showed no obvious trend with stiffness. *P<0.05, **P<0.01,***P<0.001. n=3.

When hUC-MSCs were cultured on matrices with different stiffness of 1-10kpa, 13-16 kPa, 35-38 kPa, and 62-68 kPa for 24h, the mRNA of Piezo1 increased in stiffness-dependent mode (Figure 8A), and the mRNA of Piezo2 showed no obvious tendency with stiffness by qRT-PCR. Therefore, we chose Piezo1 as object for subsequent research (Figure 8B).

**Figure 9:**
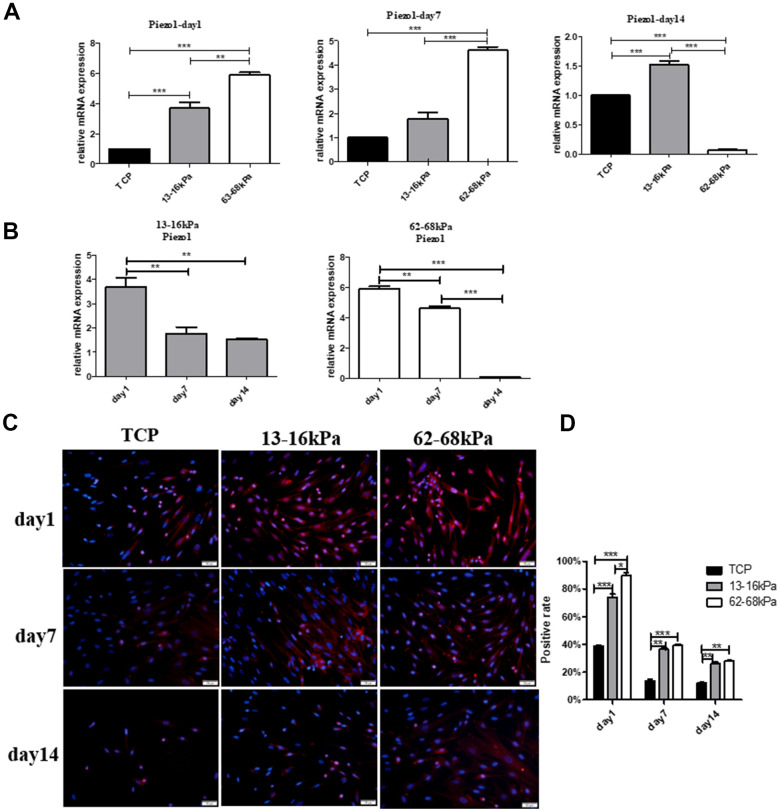
**The effect of matrix stiffness on the expression of Piezo1.** (**A**) QRT-PCR detection of Piezo1 on different matrix of 13-16 kPa, 62-68 kPa and control group at the same time point, the 1^st^ day, the 7^th^ day and the 14^th^ day. On the 1^st^ day and the 7^th^ day, Piezo1 increased with increasing matrix stiffness, and on the 14^th^ day, the expression of Piezo1 on matrix of 13-16 kPa was significantly higher than that of the other two groups. (**B**) The expression tendency of Piezo1 on the same stiffness changed with time. On the same stiffness, Piezo1 was highly expressed only on the first day. (**C**, **D**) Immunofluorescence detection in the same condition as qRT-PCR. The results also showed that compared with other time points, Piezo1 was expressed the most on the 1^st^ day, and compared with other stiffness, it was expressed most on 62-68 kPa. n=3.

Then hUC-MSCs were cultured on the matrix of 13-16 kPa and 62-68 kPa, we detected the expression of Piezo1 on the 1^st^ day, the 7^th^ day and the 14^th^ day by qRT-PCR and immunofluorescence technique. QRT-PCR results showed that at the same time point on the 1^st^ day and the 7^th^ day, Piezo1 increased with increasing matrix stiffness, and on the 14^th^ day; the expression of Piezo1 on soft matrix was significantly higher than that of the other two groups (Figure 9A). On the same stiffness, Piezo1 was highly expressed only on the first day, and Piezo1 on 13-16 kPa remained stable in subsequent times, but Piezo1 in 62-68 kPa substrates decreased gradually over time (Figure 9B). Piezo1 on the soft matrix was sensitive to matrix stiffness only on the 1^st^ day, but the sensitivity of Piezo1 on the hard matrix lasted longer. Immunofluorescence results also showed that Piezo1 expression was highest on the 1^st^ day and on 62-68 kPa, and its expression decreased over time, which was consistent with the qPCR results (Figure 9C, 9D).

**Figure 10:**
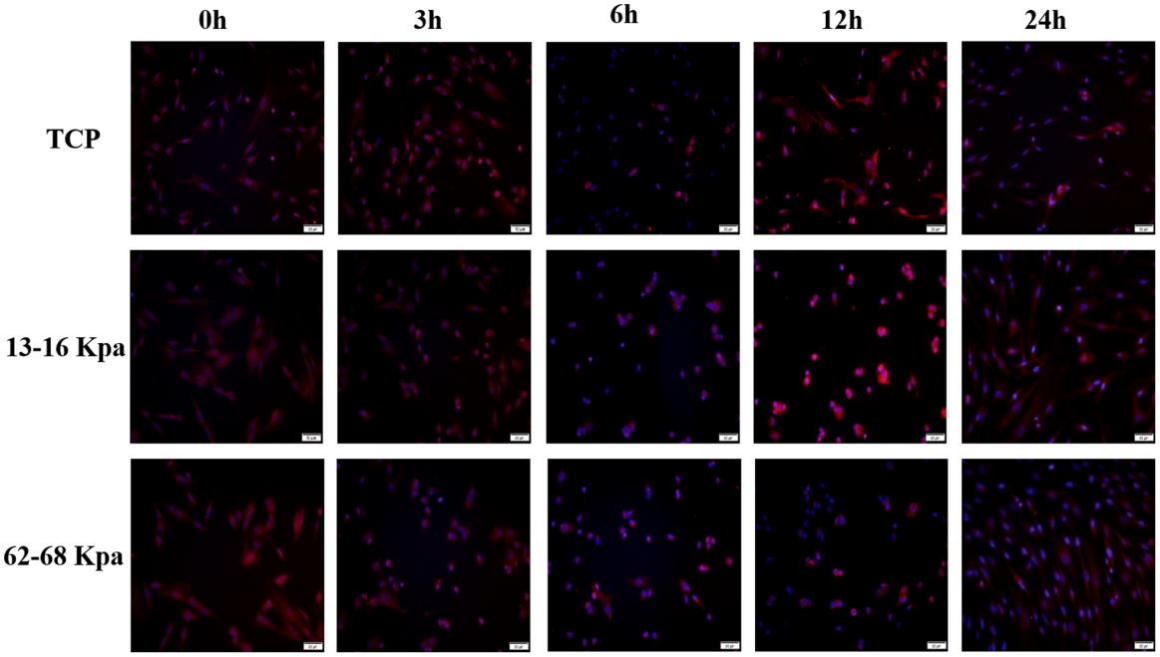
**The expression of Piezo1 was the most obvious at 12h for 13-16kpa and at 0h for 62-68kpa within 24 h.** n=3.

Since the expression position and amount of Piezo1 may be different at different time points, then immunofluorescence detection of Piezo1 at 0 h, 3h, 6h, 12h, 24h was performed. It was obvious that Piezo1 expression was highest at 12h on 13-16 kPa, and at 0 h on 62-68 kPa (Figure 10).

### The relationship among Piezo1, integrin β1 and calcium ions

**Figure 11:**
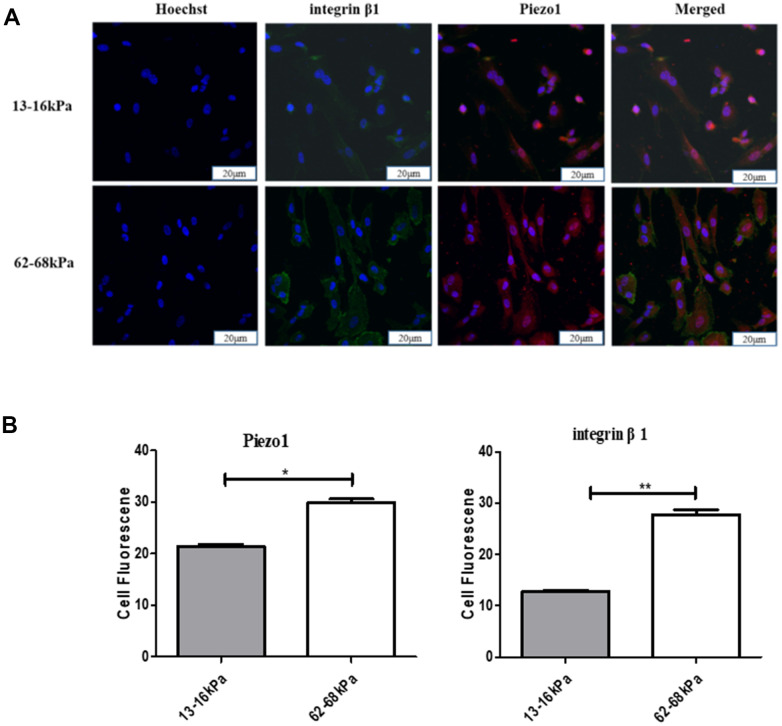
**The relationship between Piezo1 and integrin β1.** (**A**) Immunofluorescence detection of Piezo1 and integrin β1 on different matrix stiffness. (**B**) Statistical analysis of immunofluorescence intensity. *P<0.05,**P<0.01,***P<0.001. The expressions of both Piezo1 and integrin beta 1 increased with increasing stiffness, and were both highly expressed on 62-68kpa and low on 13-16kpa. n=3.

To explore the relationship between Piezo1 and integrin β1, we conducted co-stained immunofluorescence of them at 24 h on different matrix stiffness. The expression of both Piezo1 and integrin β1 increased with increasing stiffness. They were both highly expressed on 62-68 kPa and lowly expressed on 13-16 kPa (Figure 11A, 11B).

**Figure 12:**
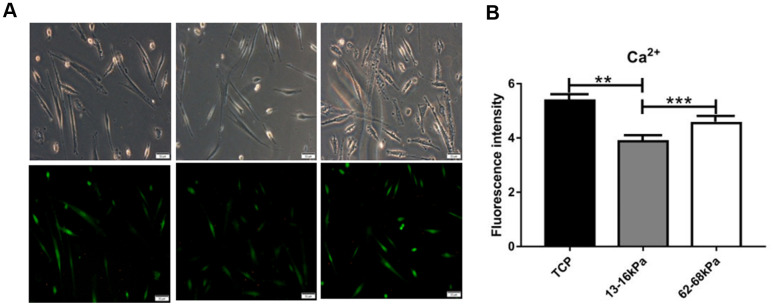
**Immunofluorescence detection of calcium ions.** (**A**) Fluorescence intensity of Calcium ions expression. (**B**) Calcium ions fluorescence intensity statistics. At 24h, the fluorescence intensity of intracellular calcium ions was the highest on the matrix of 62-68kpa and the lowest on the matrix of 13-16kpa. n=3.

Calcium ions plays an important role in the electrical microenvironment of myocardial tissue, and the increase or decrease of its concentration has a certain effect on the impulse and contraction of myocardium, which is an important functional index of cardiomyocytes’ maturation. Calcium-mediated signal transduction may take part in inducing cardiac differentiation of stem cells and regulating the maturation of differentiated cardiac cells *in vitro*. At 24h, the fluorescence intensity of intracellular calcium ions was the highest on the matrix of 62-68kpa and the lowest on the matrix of 13-16kpa (Figure 12), indicating that matrix stiffness may lead to changes in Ca^2+^ concentration. Then we knocked down Piezo1 in the hUC-MSCs, but statistical comparison revealed no significant difference on Ca^2+^ concentration.

## DISCUSSION

hUC-MSCs were chosen in this study because they have the characteristic of trauma-free extraction, sufficient source, and suitable for allograft transplantation. And some current studies *in vivo* have supported its clinical application. For myocardial infarction, therapeutic dose of 3×10^6^ cells can improve ischemic level of myocardium, and the infarct area decreased significantly and left ventricular ejection fraction increased obviously [47]. For acute myocardial infarction, 4×10^7^ cells can be used to improve myocardial perfusion and function, increase vascular density, and reduce cell apoptosis. hUC-MSC could be converted into cardiomyocytes and vascular endothelial cells [48]. For chronic myocardial ischemia, therapeutic dose of 3×10^7^ cells can improve left ventricular function, be helpful for myocardium perfusion and remodeling. Many stem cells emerged beyond the infarct area, some of which expressed von Willebrand factor [49]. All these evidences indicate that hUC-MSC transplantation is a safe and feasible treatment for cardiovascular diseases such as myocardial infarction.

The results of flow cytometric analysis and adipogenic and osteogenic induction showed that the hUC-MSCs isolated form Wharton's Jelly tissue met the minimum standards for the identification of MSCs established by the Mesenchymal and Tissue Stem Cell Committee of the International Society for Cellular Therapy [50].

The differentiation of pluripotent stem cells into cardiomyocytes goes through four stages: mesoderm, germinal mesoderm, myocardial precursor, and mature cardiomyocytes. It is divided into two phases: early differentiation and late differentiation. The early differentiation includes three stages except mature myocardial stage. The myocardial marker genes which are specifically expressed during cardiomyocyte development, can be used to evaluate cardiomyogenic differentiation [51–53]. This paper selects early myocardial markers GATA4, Nkx2.5, Mesp1 and mature myocardial markers cTnT, cTnI, α-actin as indicators of myocardial detection. qRT-PCR results showed that on 13-16 kPa, the expression of myocardial markers except cTnT, were highest ed on the 7^th^ day and the 14^th^ day. On the 13-16 kPa matrix, the expression of cTnT decreased on the 14^th^ day, while the expression of cTnI gradually increased. There may be a competitive growth relationship between cTnT and cTnI. It was reported that cTnT can be expressed in adult myocardium as well as skeletal muscle tissue under pathological conditions, cTnI only be expressed in adult myocardium [54]. But on 62-68 kPa matrix, the changes of myocardial markers were irregular. The cells on matrix stiffness of 13-16 kPa have a trend of cardiomyogenic differentiation, but the cells on matrix stiffness of 62-68 kPa have not differentiated into cardiomyocytes. It is possible that 62-68 kPa is not suitable for regulating myocardial differentiation.

Piezos, as the largest ion channels currently found, are also known to have the largest transmembrane region of all membrane proteins, consisting of two members, Piezo1 and Piezo2. Piezo1 is expressed in many tissues including endothelial cells, kidneys, lungs, and bladder [55, 56]. Our results showed that the stiffness-dependent growth of Piezo1 was observed on PAAm hydrogels with different matrix stiffness 1-10kPa, 13-16kPa, 35-38kPa and 62-68kPa, but Piezo2 doesn’t have an obvious trend with stiffness, indicating that Piezo1 is sensitive to the stimulation of matrix stiffness. The change of Piezo1 on different matrix stiffness may influence myocardial differentiation of stem cells. Studies have reported that Piezo1 play a role in vascular development, such as knockdown Piezo1 in mouse endothelial cells led to angiorrhexis during vascular remodeling and embryonic death [57], indicating that Piezo1 is involved in cardiovascular development and has a regulatory effect on cell differentiation. Piezo2 is involved in physiological process, such as proprioception, light touch, and pain. The mechanism is that mechanical stimulation sensed by cell membrane leads to Piezo2 activation, and it is translated into electrical signals quickly to produce a mechanically sensitive current, thus Piezo2 participates in the regulation of physiological processes in the body [58].

It is found that Piezo1 has close relationship with integrin β1. For example, knockdown Piezo1 of lung epithelial cells led to inactivation of integrin, decrease of cytoplasmic calpain activity, and decrease of cell adhesion [40]. Furthermore, decrease of Piezo1 expression was also detected in other cancer cells, such as thyroid cancer cells and gastric cancer cells [59, 60], which may be related to low adhesion and easy migration of cancer cells. Above all, Piezo1 knockdown and integrin inactivation can reduce cell adhesion and promote migration. In addition, another study found that almost all the cations of the cells could flow in intracellular, such as sodium, potassium, calcium, magnesium, but Piezo1 particularly prefer to mediate calcium ions influx [61]. Therefore, it is necessary to explore the relationship among Piezo1, integrin β1 and calcium ions in the process of matrix stiffness regulating myocardial differentiation of stem cells.

In this experiment, Piezo1 expression at 24 h was significantly higher than the 7^th^ day and the 14^th^ day. And Piezo1 expression on the 13-16kPa was lower than 62-68 kPa. On the 1^st^ day, the expression of Piezo1 changed at different time points. Piezo1 is more sensitive to matrix stiffness within 24 h, hence 24 h was selected as the time point for research. Our study found that both the expression of integrin β1 and calcium ions on 13-16 kPa were lower than that on 62-68 kPa at 24 h, and there is a positive correlation between Piezo1 and matrix stiffness.

Piezo1, integrin β1 and calcium ions have similar variation trend with matrix stiffness. We speculate that the matrix stiffness of 13-16 kPa changes the mechanical force sensed by cell membrane, and then decreases cell adhesion and the expression of integrin β1. Integrin β1 interacts with ECM outside the cell and with cytoskeleton inside the cell. The changes of cytoskeleton structure inhibit the mechanically sensitive cation channel Piezo1, so that fewer calcium ions enter the cytoplasm and regulate the differentiation into cardiomyocytes. The result is different on the matrix stiffness of 62-68 kPa.

However, after knocking down Piezo1, the variation of Ca^2+^ concentration of hUC-MSCs with stiffness disappeared. We infer that the concentration of calcium ions in the hUC-MSCs was partly influenced by Piezo1, but many other signal transduction pathways also affect the Ca^2+^ concentration. When a cell receives a mechanical stimulus, it can activate the release of Ca^2+^ from storage compartments within the cell, such as the endoplasmic reticulum (ER). Ca^2+^ can also enter the cell from outside via channels located in the plasma membrane. The Ca^2+^ influx at the plasma membrane and release from ER are the only two sources for Ca^2+^ oscillations in HMSCs [62]. A research found lowering the matrix stiffness to 1 kPa significantly inhibited both the magnitudes and frequencies of the cytoplasmic Ca^2+^ oscillation in comparison to stiffer or rigid substrate. This Ca^2+^ oscillation was shown to be dependent on ROCK, a downstream effector molecule of RhoA, but independent of actin filaments, microtubules, myosin light chain kinase, or myosin activity [63]. Another study found the active actomyosin contractility plays an important role in Ca^2+^ influx. This ER Ca^2+^ release upon mechanical stimulation is mediated not only by the mechanical support of cytoskeleton and actomyosin contractility, but also by mechanosensitive Ca^2+^ permeable channels on the plasma membrane, specifically TRPM7. However, Ca^2+^ influx at the plasma membrane via mechanosensitive channels is only mediated by the passive cytoskeletal structure. Thus, active actomyosin contractility is essential for the response of ER to the external mechanical stimuli, distinct from the mechanical regulation at the plasma membrane [62]. We speculate that both Ca^2+^ and mechanically sensitive ion channel are redundant, and Piezo1 plays a limited role in regulating Ca^2+^ concentration.

## CONCLUSIONS

hUC-MSCs tend to differentiate into the myocardium on the matrix stiffness of 13-16kPa, compared with matrix stiffness of 62-68kPa. The differentiation into the myocardium is related to the relatively low expression of Piezo1, integrin β1 and Ca^2+^ concentration on soft matrix.

## MATERIALS AND METHODS

### Generation of polyacrylamide gels with different stiffness

The glass cover slips were treated with 3-aminopropyltrimethoxysilane and 0.5% glutaraldehyde. Then, 8% acrylamide (sigma, USA) was mixed with varying concentrations of bisacrylamide (0.1% and 0.7%) (Sigma, USA). Polymerization was initiated with N,N,N′,N′-tetramethylethylenediamine (TEMED) and ammonium persulfate (sigma, USA). Then, 0.2 mg/mL N-sulfosuccinyimidyl-6-(4′-azido-2′-nitrophenylamino) hexanoate (sulfo-SANPAH) (Thermo, USA) dissolved in 10 mM HEPES (pH 8.5) was applied to cover polyacrylamide (PAAM) gel and exposed to 365 nm ultraviolet light for 70 min for photo activation in 24-well plates. The PAAm sheet was washed for three times with PBS to remove excess reagent and incubated with fibronectin solution (1 μg/cm^2^; Sigma, USA) overnight at 4° C. Before the cells were plated, the PAAm matrices were soaked in PBS and then in DMEM at 4° C. The Young’s modulus of the PAAm hydrogels was quantified using a biomechanical testing machine under contact load at a strain rate of 0.5 mm/s [45].

### Cell culture

Primary hUC-MSCs were isolated from the human umbilical cord of 4 different human subjects. It was approved by the ethics committee of Jilin University and conformed to the regulatory standards. Washed the umbilical cord with saline solution and maintained in Phosphate Buffer Saline (PBS) (Beijing Dingguo Biotech Co., Ltd, China) consisting of 1% penicillin/streptomycin (Dalian Meilun Biotech Co., Ltd, China). Cut it into fragments of about 2-3 cm in length, removed arteries and veins from tissue slices. Cut them into small pieces in the area of 2 mm^3^. Attached these small pieces to the six-hole plate evenly about 5 mm apart. After 5-10 minutes, added a drop of medium consisting of 10% fetal bovine serum (Gibco, USA) supplemented with 1% penicillin/streptomycin on every piece and incubated them in an atmosphere of 5% CO_2_ at 37° C for 4-6 hours. After subculture, the P5 to P7 generation cells were seeded on the PAAm hydrogels of 13-16 kPa and 62-68 kPa.

### Flow cytometric analysis

Expression of MSC surface markers was determined using flow cytometry and immunofluorescence staining. Cells were collected, washed thrice with phosphate buffered saline (PBS), and fixed with 4% polyformaldehyde for 20 min. The cells were then blocked with 1% BSA in PBS for 30 min and incubated with 10 μg/mL anti-CD34, anti-CD44, anti-CD45, anti-CD90, and anti-CD105 mAbs (eBioscience, USA) for 1 h [46].

### Adipogenic and osteogenic induction

When the cells were fused to 80-90%, replaced the culture medium with adipogenic and osteogenic induction solution respectively. We conducted adipogenic induction for 2 weeks and osteogenic induction for 4 weeks. For evaluation of lipid droplets, the cells were fixed with 4% paraformaldehyde for 10 min, stained with oil red O for 10 min at room temperature and decolorized with 70% isopropanol. For characterization of the calcium nodules, the cells were fixed with 3.7% paraformaldehyde and stained with 1% of alizarin red for 20 min at room temperature. The cells were observed under an inverted phase contrast microscope.

### Gene expression analysis

Quantitative real-time reverse transcription polymerase chain reaction (qRT-PCR) was used to determine the relative gene expression of early myocardial markers Nkx2.5, GATA4, Mesp1 and mature myocardial markers cTnT, cTnI, α-actin.

Total RNA was extracted by the TRI reagent (Invitrogen, USA). The same amount of total RNA was used to synthesize the first strand cDNA with the PrimeScript^TM^ RT reagent kit (Takara Bio Inc, Japan). We need to remove the gDNA at first. The mixture needs to be mixed evenly and be incubated in metal bath at the temperature of 42° C for 2 minutes. Then we obtained the cDNA through the reverse transcription reaction, which consisted of 37° C for 15min, 85° C for 5s and 4° C for 5min.

The real-time transcription polymerase chain reaction mix contained a 20 ng template of cDNA and 400 nM each of the forward and reverse primers (Sangon Biotech Co., Ltd, China) using the SYBR *Premix Ex* Taq (Takara Bio Inc, Japan). Primer sequences for the amplification are shown in Table 1. Every gene had more than 3 paralleled holes.

**Table 1:** Primers.

**Gene name**	**Forward(5’to 3’)**	**Reverse(5’to 3’)**
Piezo1	CATCTTGGTGGTCTCCTCTGTCT	CTGGCATCCACATCCCTCTCATC
Piezo2	CACCTGGCTACAACTGCTCA	CCCGATGTCAGGTACAAACA
GATA4	AGAAGGCAGAGAGTGTGTCA	CAGTGTGGTGGTGGTAGTCT
Nkx2.5	CAAGGACCCTAGAGCCGAAA	TCAAGGCGCTGGAGAACAA
cTnT	AGCATCTATAACTTGGAGGCAGAG	AGGAGTTCAATCACTTGGCG
cTnI	AATCTAAGATCTCCGCCTCG	TCAGATCTGCAATCTCCGTG
Sox2	CGCCCCCAGCAGACTTCACA	CTCCTCTTTTGCACCCCTCCCATTT
	AGAAGGATGTGGTCCGAGTGTG	CCACCCTTTGTGTTCCCAATTCC
Oct4	GAAGGTGAAGGTCGGAGTCAAC	CAGAGTTAAAAGCAGCCCTGGT
GAPDH	CCTGAGGAGCCCAAGTGACA	GAAGGTGCTGAGGCCAAAAAG
Mesp1 α-actin	AAGATCAAGATCATTGCTCCTC	GGACTCATCGTACTCCTG

Primer sequences of early myocardial markers and mature myocardial markers used in q-RT-PCR are shown in Table 1.

The PCR thermal profile consisted of 50° C for 2 minutes and 95° C for 30 seconds, followed by 40 cycles of 95° C for 10 seconds and 60° C for 30 seconds, and finally, 95° C for 15 seconds, 60° C for 1 minute and 95° C for 15 seconds. Genes were normalized to the housekeeping gene GAPDH, and fold differences were calculated using the comparative Ct method.

### Immunofluorescence staining

Cells on PAAm hydrogels were fixed with 4% paraformaldehyde for 15 minutes at room temperature. Following blocking for 1 h in 5% fetal bovine serum, substrates were incubated with primary antibodies for 12 h at 4° C, and with secondary antibody selected according to the species of primary antibodies for 1 h at room temperature. Then cells were stained by Hoechst diluted with PBS (1:1000) at room temperature for 5 minutes. Images were acquired with an inverted fluorescence microscope (Olympus, Japan).

### Western blot

Extracted total proteins from collected cells by protein lysis solution (RIPA: PMSF=100:1). Centrifuge the lysate at 4° C, 12000 rmp for 30 min. Used the BCA kit to measure protein concentration. Boiled protein with Loading Buffer for proteins denaturation. According to the molecular weight of protein to select the appropriate gel ratio to configure the electrophoresis gel. This experiment mainly used the 12% separation gel, 8% separation gel, and the 5% concentrated gel, as shown in the Table 2. When gels have been prepared, assembled electrophoresis tank and added electrophoresis buffer to inner core over short plate. The protein Marker and samples were added in the holes of the gels. Electrophoresis parameter were set as 80 V for 30 minutes and 100 V for 90 minutes. The wet transfer method was used. Placed the completed transfer film instrument on the ice, and the transfer printing parameters were set as 100 V for 1-1.5 hours. Following blocking for 1 h in 5% skim milk powder and placing on shaker. Substrates were incubated with primary antibodies diluted by TBST for 12 h at 4° C, and with secondary antibody for 1 h at room temperature. Washed by TBST for 3 times. The ECL hypersensitive chromogenic solution mixing the solution A with solution B (1:1) was used to detect the signal.

**Table 2:** Solutions for preparing gels for electrophoresis.

**Reagent**	**5% concentrated gel (2ml)**	**8% separation gel (5ml)**	**12% separation gel (5ml)**
ddH2O	1.4ml	2.3ml	1.6ml
30%Acr	330μl	1.3ml	2.0ml
Tri-Hcl (PH8.8)		1.3ml	1.3ml
Tri-Hcl (PH6.8)	250μl		
10%SDS	20μl	50μl	50μl
10%APS	20μl	50μl	50μl
TEMED	2μl	4μl	4μl

Ingredients of the 12% separation gel, 8% separation gel, and the 5% concentrated gel are shown in the Table 2.

### Calcium ion fluorescent probe staining

Cells were washed with PBS for three times. Substrates were incubated with 0.5μM of Fluo-4AM working fluid (Solarbio, China) for 30 minutes at 37° C. Then washed by PBS for three times again and the change of calcium ion concentration was detected by fluorescence microscope.

### Statistical analysis

We did more than 3 independent experiments for every step, and the statistic and analysis were based on the results of experiments for more than 3 times. The aspect ratio and area were measured for cells in 3 paralleled holes (n=3). The qRT-PCR and Western Blot were conducted for 3 independent experiments (n=3). The immunofluorescence staining and calcium ion fluorescent probe staining were done for 3 independent experiments (n=3).

Data are expressed as the mean ± standard deviation (SD). Statistical analyses were performed using the statistics package SPSS 13.0 (SPSS, Chicago, IL, USA). A comparison among all groups was carried out using one-way analysis of variance. A p-value less than 0.05 was considered statistically significant. Then we use Tukey’s multiple comparisons test to compare the data between every two groups.
